# Dual Benefits of Oral Tranexamic Acid: Reducing Melasma Severity and Inflammation

**DOI:** 10.1111/jocd.70257

**Published:** 2025-05-20

**Authors:** Abdullah Demirbas, Gozde Ulutas Demirbas, Esin Diremsizoglu, Mustafa Esen

**Affiliations:** ^1^ Kocaeli University Faculty of Medicine Department of Dermatology Kocaeli Turkey; ^2^ Kocaeli City Hospital Department of Dermatology Kocaeli Turkey; ^3^ Dicle University Faculty of Medicine Department of Dermatology Diyarbakır Turkey

**Keywords:** high‐density lipoprotein (HDL), inflammatory markers, lymphocytes, melasma, monocytes, neutrophils, skin pigmentation disorders, tranexamic acid

## Abstract

**Background:**

Melasma is a chronic hyperpigmentation disorder influenced by hormonal factors, ultraviolet exposure, and inflammation. While oral tranexamic acid (TXA) is an established treatment, its effects on systemic inflammation remain unclear.

**Aims:**

This study aimed to evaluate the impact of TXA on melasma severity and inflammatory markers.

**Methods:**

This retrospective study included 80 melasma patients and 80 healthy controls. Patients received oral TXA (500 mg/day) for 3 months. Melasma severity was assessed using the Melasma Area and Severity Index (MASI), and inflammatory markers (monocyte, neutrophil, lymphocyte, HDL, MHR, MLR, NLR) were measured at baseline, Month 1, and Month 3. Changes within the melasma group and comparisons with controls were analyzed.

**Results:**

At 3 months, melasma severity significantly improved, with a 65.1% reduction in MASI (from 12.9 to 4.5, *p* < 0.001). Monocyte, neutrophil, MHR, MLR, and NLR levels significantly decreased, while HDL and lymphocyte levels increased (*p* < 0.001). Compared to controls, baseline inflammatory marker levels differed significantly; however, at month 3, only monocyte, MHR, and HDL remained significantly different (*p* < 0.05). Regression analysis identified NLR and HDL as significant predictors of melasma severity reduction (*p* = 0.045 and *p* = 0.011, respectively).

**Conclusion:**

Oral TXA not only improved melasma severity but also modulated systemic inflammation. The association between NLR, HDL, and treatment response suggests their potential as biomarkers for monitoring therapeutic efficacy.

## 1 Introduction

Melasma is a common hyperpigmentation disorder characterized by dark, irregular facial patches. It predominantly affects women of reproductive age and is often triggered by ultraviolet (UV) radiation, hormonal fluctuations such as pregnancy, oral contraceptives, and photosensitizing medications [1]. Clinically, melasma presents in centrofacial, malar, and mandibular distribution patterns, and it significantly impacts patients' quality of life, causing psychological distress and social stigmatization [2, 3].

Emerging evidence suggests that chronic inflammation may contribute to melasma pathogenesis. Prolonged UV exposure induces cutaneous inflammation, leading to fibroblast activation, increased stem cell factor (SCF) expression, and subsequent melanogenesis [4, 5, 6]. Systemic inflammatory markers, such as monocyte‐to‐HDL ratio (MHR), monocyte‐to‐lymphocyte ratio (MLR), and neutrophil‐to‐lymphocyte ratio (NLR), have been investigated in various dermatological conditions, including acne, hidradenitis suppurativa, psoriasis, and vitiligo [7, 8, 9, 10, 11]. However, their role in melasma remains underexplored, and limited studies have evaluated whether systemic inflammatory markers correlate with disease severity or response to treatment.

Oral tranexamic acid (TXA) has gained attention as an effective treatment for melasma due to its melanogenesis‐inhibiting and anti‐inflammatory properties. TXA reduces melanin synthesis by inhibiting plasminogen activation, thereby lowering melanocyte‐stimulating hormone (MSH) levels. Additionally, TXA modulates inflammation by suppressing mast cell activation and fibroblast‐derived growth factors while also reducing vascular endothelial growth factor (VEGF) and endothelin‐1 levels [4]. While previous studies have focused on TXA's effects on pigmentation, its impact on systemic inflammatory markers, particularly monocyte, lymphocyte, neutrophil, and HDL levels, remains unclear [2, 12, 13, 14, 15, 16, 17, 18, 19].

This study aimed to assess the impact of oral TXA on melasma severity and systemic inflammatory markers, including MHR, MLR, and NLR, by comparing patients with melasma to a healthy control group. Inflammatory markers were evaluated before and after treatment to determine their association with treatment response and their potential role as indicators of therapeutic efficacy.

## 2 Methods

This retrospective study included 80 female patients diagnosed with melasma and 80 age‐matched healthy female controls, analyzed between 2019 and 2021. The control group consisted of healthy female healthcare workers with no known systemic or dermatological diseases. Patients with melasma were required to have a dermatologist‐confirmed diagnosis and no prior treatment within the last 6 months. Exclusion criteria included pregnancy or lactation, autoimmune or systemic diseases, and the use of anticoagulants or other medications affecting inflammatory markers.

Patients in the melasma group received oral TXA (500 mg/day) for 3 months, with evaluations conducted at baseline (Month 0), Month 1, and Month 3. The control group did not receive any treatment and was assessed at a single time point for comparison.

Melasma severity was assessed using the Melasma Area and Severity Index (MASI), which evaluates four facial regions: forehead, right and left malar regions, and chin. The extent of involvement (%), darkness (0–6), and homogeneity (0–4) were recorded, and the total MASI score was calculated using a weighted formula: Score = [Area (%) × Darkness × Homogeneity], applying region‐specific multipliers: 0.3 for the forehead, 0.3 for each malar region, and 0.1 for the chin.

Inflammatory markers, including monocytes, neutrophils, lymphocytes, and HDL, were measured at baseline, Month 1, and Month 3. The ratios of these markers—MHR (monocyte‐to‐HDL ratio), MLR (monocyte‐to‐lymphocyte ratio), and NLR (neutrophil‐to‐lymphocyte ratio) were calculated to assess systemic inflammatory changes.

### 2.1 Statistical Analysis

Normality was assessed using the Shapiro–Wilk test. Parametric variables were analyzed using paired t‐tests and repeated measures ANOVA, while non‐parametric data were evaluated with the Friedman test. Linear regression analysis was performed to examine the association between changes in melasma severity (ΔMASI) and inflammatory markers (ΔNLR, ΔMLR, ΔHDL, etc.) over time. A *p* value of < 0.05 was considered statistically significant.

### 2.2 Ethics Statement

This study was conducted in accordance with the Declaration of Helsinki and approved by the institutional ethics committee (Decision Date, Number: 2021/09‐17). Written informed consent was obtained from all participants prior to study enrollment.

## 3 Results

A total of 160 female participants were included in the study, consisting of 80 patients diagnosed with melasma and 80 healthy controls. The median age of the melasma group was 39 years (IQR: 36–46) and the median disease duration in the melasma group was 6 years (IQR: 3–10.75), ranging from 1 to 25 years. The control group had a median age of 29.5 years (IQR: 25.25–36).

**FIGURE 1:**
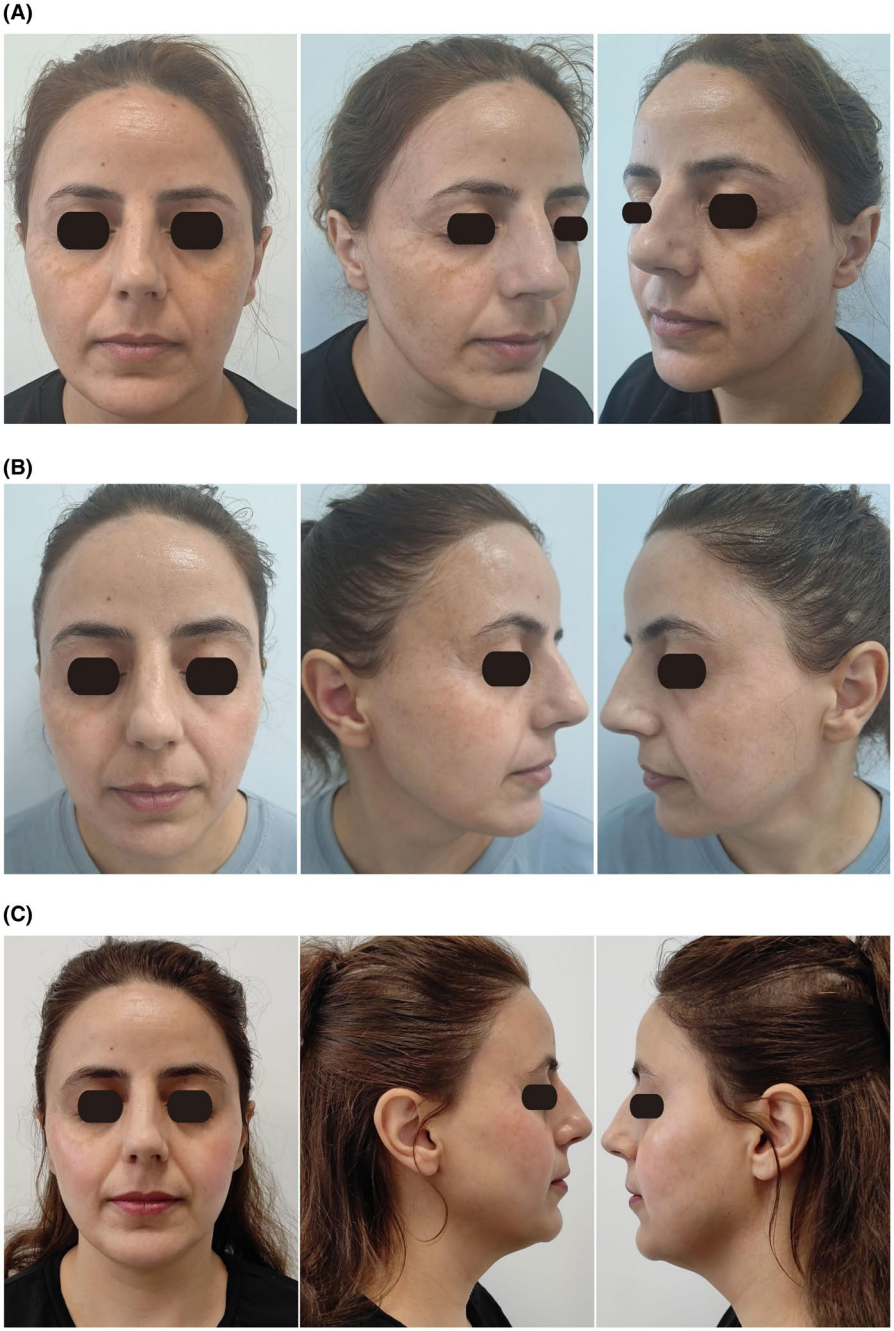
Clinical improvement in a melasma patient over time. (a) Baseline clinical presentation. (b) Clinical appearance at Month 1. (c) Clinical appearance at Month 3.

At the 3‐month follow‐up, patients receiving oral TXA showed significant improvements in melasma severity, as indicated by a reduction in Total MASI Score from 12.9 (IQR: 7.3–14.1) at baseline to 4.5 (IQR: 3.5–5) (*p* < 0.001, Table 1). Additionally, facial involvement significantly decreased, with reductions observed in the forehead (from 35% to 12%), right malar region (from 50% to 17%), left malar region (from 45% to 20%), and chin (from 10% to 5%) (*p* < 0.001). Clinical images demonstrating the patient's improvement are presented in Figure 1a–c, corresponding to baseline, Month 1, and Month 3, respectively.

**TABLE 1:** Comparison of baseline and 3‐month post‐treatment clinical variables in melasma patients.

Variable	Baseline (0), median (IQR)	Month 3, median (IQR)	*p* *
Forehead (%)	35 (25–45)	12 (10–15)	< 0.001
Right malar (%)	50 (35–55)	17 (15–17)	< 0.001
Left malar (%)	45 (40–55)	20 (18–21)	< 0.001
Chin (%)	10 (10–15)	5 (2–6)	< 0.001
Pigmentation severity	5 (4–6)	2 (2–2)	< 0.001
Homogeneity	3 (2–4)	1 (1–2)	< 0.001
Total MASI score	12.9 (7.3–14.1)	4.5 (3.5–5)	< 0.001

Abbreviation: MASI, Melasma Area and Severity Index.

* The Wilcoxon signed‐rank test was used to compare baseline and post‐treatment values within the melasma group.

**FIGURE 2:**
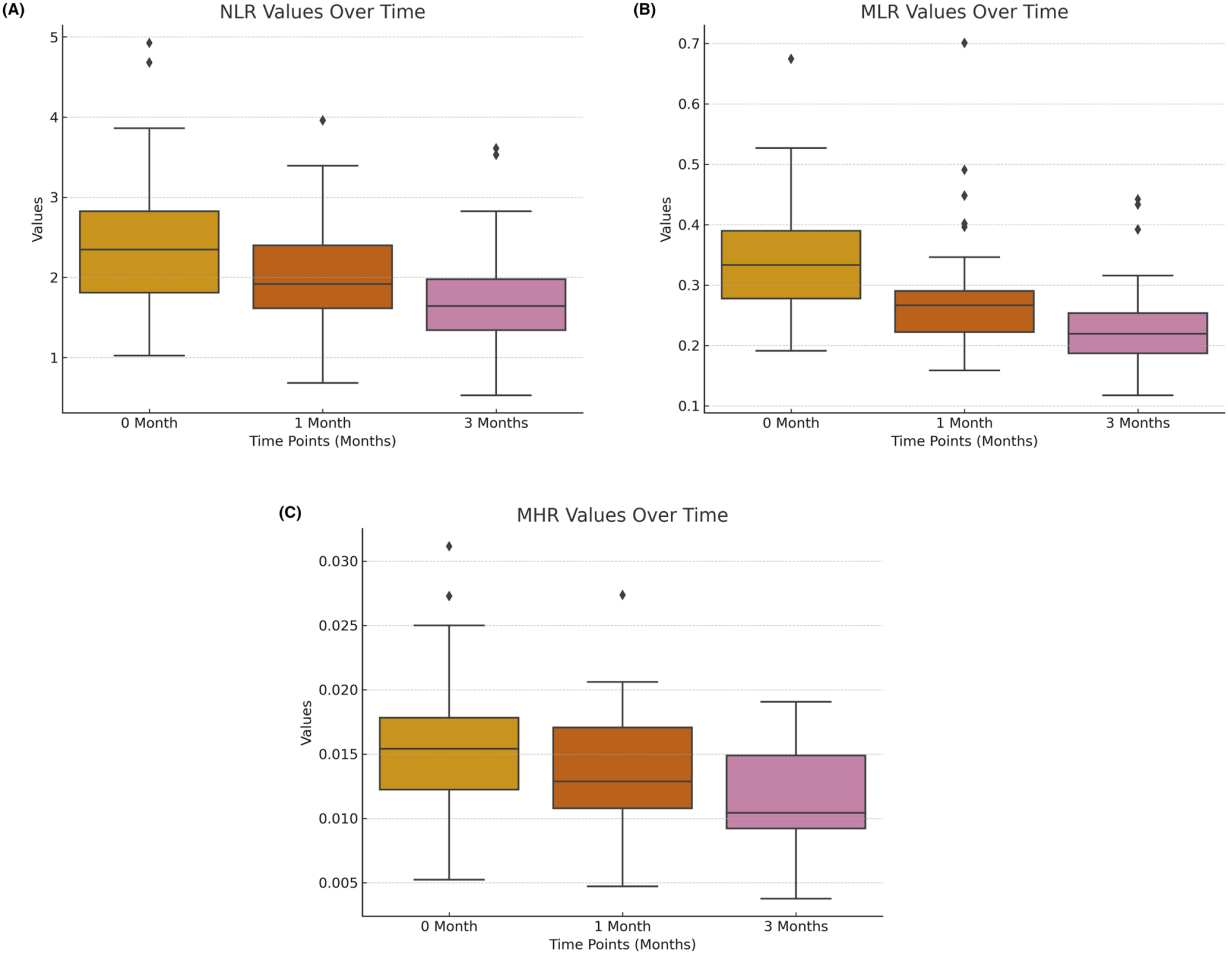
Box plots of inflammatory marker changes over time. (a) Box plot of NLR values at baseline (0 month), Month 1, and Month 3. (b) Box plot of MLR values at baseline (0 month), Month 1, and Month 3. (c) Box plot of MHR values at baseline (0 month), Month 1, and Month 3.

Inflammatory markers showed significant changes over time. At baseline, monocyte, MHR, and NLR levels were significantly higher in the melasma group compared to controls, while lymphocyte and HDL levels were significantly lower (*p* < 0.001, Table 2). At the 3‐month follow‐up, monocyte and neutrophil levels had significantly decreased, whereas HDL and lymphocyte levels had increased (*p* < 0.001). Additionally, MHR, MLR, and NLR levels showed a significant reduction over time (*p* < 0.001, Table 2, Figure 2a–c). When comparing the melasma group to controls at Month 3, lymphocyte, neutrophil, MLR, and NLR levels were no longer significantly different from the control group (*p* > 0.05). In contrast, monocyte and MHR levels remained significantly higher, while HDL levels remained significantly lower in the melasma group compared to controls (*p* < 0.05, Table 2).

**TABLE 2:** Comparison of inflammatory markers between control and melasma groups at baseline, Month 1, and Month 3.

Marker	Control, median (IQR)	Baseline (0), median (IQR)	*p* * (control vs. baseline)	Month 1, median (IQR)	Month 3, median (IQR)	*p* * (control vs. Month 3)	*p* ** (Month 0 vs. 1 vs. 3)
Neutrophil	3.66 (3.34–4.67)	4.30 (3.58–5.12)	0.007	3.91 (3.50–4.86)	3.67 (3.08–4.06)	0.561	< 0.001
Lymphocyte	2.08 (1.76–2.59)	1.90 (1.53–2.30)	0.003	2.12 (1.68–2.43)	2.28 (1.93–2.53)	0.236	< 0.001
Monocyte	0.480 (0.38–0.61)	0.584 (0.50–0.69)	< 0.001	0.500 (0.47–0.63)	0.500 (0.40–0.55)	< 0.001	< 0.001
HDL	50.50 (45.30–53.50)	40.00 (35.00–45.00)	< 0.001	42.00 (36.00–46.50)	45.00 (39.25–48.00)	< 0.001	< 0.001
NLR	1.6472 (1.50–2.43)	2.3452 (1.79–2.84)	< 0.001	1.9167 (1.59–2.41)	1.6438 (1.32–1.98)	0.096	< 0.001
MLR	0.2339 (0.1873–0.2805)	0.3333 (0.2778–0.3895)	< 0.001	0.2667 (0.2222–0.2899)	0.2192 (0.1869–0.2535)	0.297	< 0.001
MHR	0.0095 (0.0082–0.0118)	0.0154 (0.0122–0.0179)	< 0.001	0.0129 (0.0107–0.0171)	0.0104 (0.0091–0.0149)	0.026	< 0.001

Abbreviations: MHR, Monocyte to HDL Ratio; MLR, Monocyte to Lymphocyte Ratio; NLR, Neutrophil to Lymphocyte Ratio.

* The Mann–Whitney *U* test was used for comparisons between the control and melasma groups.

** Friedman test was used to assess differences within the melasma group across baseline, Month 1, and Month 3.

Within the melasma group, monocyte, neutrophil, MHR, MLR, and NLR levels significantly decreased, while HDL and lymphocyte levels significantly increased from baseline to Month 3 (*p* < 0.001 for all, Table 2).

Linear regression analysis revealed that ΔNLR and ΔHDL were significantly associated with ΔMASI. In the univariate model, ΔNLR (*β* = 1.794, 95% CI: 0.508–3.080, *p* = 0.001) and ΔHDL (*β* = −0.320, 95% CI: −0.514 to −0.126, *p* = 0.001) showed significant associations. In the multivariate model, both ΔNLR (*β* = 1.222, 95% CI: 0.601–2.843, *p* = 0.045) and ΔHDL (*β* = −0.249, 95% CI: −0.472 to −0.026, *p* = 0.011) remained significant (Table 3).

**TABLE 3:** Linear regression analysis of melasma severity (MASI) and changes in inflammatory markers.

Variable	Univariate *β* (95% CI)	*p*	Multivariate *β* (95% CI)	*p*
ΔNeutrophil	0.063 (−0.921–1.047)	0.898	—	—
ΔLymphocyte	−1.991 (−4.993–1.011)	0.051	—	—
ΔMonocyte	−3.713 (−12.865–5.439)	0.428	—	—
ΔHDL	−0.320 (−0.514 – −0.126)	**0.001**	−0.249 (−0.472 – −0.026)	**0.011**
ΔNLR	1.794 (0.508–3.080)	**0.001**	1.222 (0.601–2.843)	**0.045**
ΔMLR	15.212 (5.578–24.846)	**0.008**	5.079 (−6.359–16.517)	0.427
ΔMHR	27.652 (−148.603–203.907)	0.853	—	—

*Note:* Δ indicates the absolute difference of each parameter from baseline to Month 3. Bold values indicate statistical significance at *p* < 0.05.

Abbreviations: HDL, High‐Density Lipoprotein; MASI, Melasma Area and Severity Index; MHR, Monocyte‐to‐HDL Ratio; MLR, Monocyte‐to‐Lymphocyte Ratio; NLR, Neutrophil‐to‐Lymphocyte Ratio.

Among 80 patients, mild adverse effects were observed in seven patients, with headache reported in six and nausea in one.

## 4 Discussion

Melasma is a chronic hyperpigmentation disorder influenced by hormonal factors, UV exposure, and inflammation [1]. It most commonly affects women of reproductive age, typically emerging in the third and fourth decades of life, as seen in our study, where the median age of melasma patients was 39 years [2]. The median disease duration was 6 years in our study, consistent with previous reports describing melasma as a long‐standing and persistent condition in many patients [3, 4].

TXA is a plasmin inhibitor with both anti‐inflammatory and depigmenting effects, making it a promising therapeutic option for melasma. Its mechanism of action involves inhibiting melanin production and improving vascular stability. Studies have shown that TXA reduces fibroblast growth factor levels, mast cell proliferation, and vascular proliferation, thereby limiting UV‐induced melanocyte activation [12]. Additionally, TXA has been shown to prevent relapses after conventional melasma treatments. The clinical efficacy of oral TXA is well documented. A randomized study reported that 50% of patients receiving oral TXA showed clinical improvement, compared to only 5.9% in the control group [13]. A meta‐analysis further confirmed significant reductions in MASI scores over 4, 8, 12, and 16 weeks of treatment [14]. Another study with 25 patients observed a 69% reduction in MASI scores after approximately 3.7 months of treatment, reinforcing the role of TXA as an effective adjunct in refractory melasma [15]. A systematic review of 22 studies with 1280 patients concluded that oral TXA resulted in the greatest improvement in melasma severity compared to topical or intralesional administration [16]. Based on our findings, oral TXA significantly reduced melasma severity, with the total MASI score decreasing by 65.1% from baseline to month 3. Facial involvement also showed a notable reduction, particularly in the right malar (66%), left malar (55.6%), and forehead (65.7%) regions. Despite mild side effects such as gastrointestinal discomfort and menstrual irregularities, TXA is generally well tolerated and safe [12]. In our study, mild adverse effects were observed in seven patients, with headache reported in six and nausea in one.

Inflammation in melasma has received increasing attention in recent years. Histopathological studies have demonstrated increased vascular proliferation in melasma lesions, which may manifest as subclinical or visible erythema. This vascularity is thought to contribute to local endothelial and inflammatory cell recruitment, subsequently enhancing melanogenesis [17]. Elevated levels of inflammatory cytokines and chemokines in melasma patients further suggest that inflammation not only contributes to the pathogenesis but also influences disease severity [5]. Studies indicate that inflammatory conditions may exacerbate pigmentation disorders, reinforcing the importance of addressing inflammation as part of melasma management [6].

Neutrophils and monocytes are central players in the inflammatory response, while lymphocytes and HDL have anti‐inflammatory roles [7]. Prior studies have recognized MHR, MLR, and NLR as useful inflammatory markers in various dermatological diseases. Elevated MHR and MLR levels have been linked to increased oxidative stress and inflammation in conditions such as acne and vitiligo, while higher NLR levels have been associated with disease severity in psoriasis [7, 8, 9]. In Behçet's disease, reductions in MHR, MLR, and NLR following treatment with colchicine highlight their potential as indicators of disease activity and response to anti‐inflammatory therapies [10]. Similarly, in hidradenitis suppurativa, systemic inflammatory markers correlate with disease severity, emphasizing their relevance in dermatological inflammation [11].

In our study, baseline inflammatory marker levels significantly differed between melasma patients and controls, with higher monocyte, MHR, and NLR levels and lower lymphocyte and HDL levels in the melasma group. After TXA treatment, monocyte and neutrophil levels decreased, while HDL and lymphocyte levels increased, suggesting a potential anti‐inflammatory effect. Additionally, MHR, MLR, and NLR levels declined, reinforcing the role of TXA in modulating inflammation. By Month 3, lymphocyte, neutrophil, MLR, and NLR levels were no longer significantly different from controls. However, monocyte and MHR levels remained elevated, while HDL remained lower, suggesting persistent inflammatory alterations. These findings highlight the relevance of inflammatory markers in monitoring treatment response and suggest that TXA may exert anti‐inflammatory effects beyond its depigmenting properties.

Importantly, our findings revealed a significant association between inflammatory marker changes and melasma severity reduction. Linear regression analysis showed that greater reductions in NLR and increases in HDL were correlated with greater MASI improvement. These results support the hypothesis that systemic inflammation contributes to melasma pathogenesis and that NLR and HDL may serve as potential biomarkers for treatment efficacy. While MHR and MLR have been linked to inflammatory skin diseases, they were not significantly associated with melasma severity changes in our study.

Our study has some limitations. It is a non‐randomized study with a moderate sample size, requiring larger cohorts with long‐term follow‐up to confirm findings. Additionally, a limited number of inflammatory markers were analyzed, and as all participants were female, the results may not be generalizable to male patients.

In conclusion, our study demonstrated that oral TXA significantly reduced melasma severity, with a 65.1% decrease in the total MASI score over 3 months. Additionally, inflammatory markers showed notable changes following treatment; monocyte, neutrophil, MHR, MLR, and NLR levels significantly decreased, while HDL and lymphocyte levels increased. These findings suggest that TXA not only improves pigmentation but also modulates systemic inflammation. Moreover, NLR and HDL were significantly associated with treatment response, highlighting their potential as biomarkers for monitoring therapeutic efficacy in melasma patients. Further studies are needed to confirm these findings and assess long‐term outcomes.

## Ethics Statement

The study received approval from the institutional ethics committee (Decision Date, Number: 2021/09‐17).

## Consent

Written informed consent was obtained from all participants prior to study enrollment.

## Conflicts of Interest

The authors declare no conflicts of interest.

## Data Availability

The data that support the findings of this study are available from the corresponding author upon reasonable request.
